# Solitary contralateral adrenal metastasis of renal cell carcinoma 15 years following radical nephrectomy: A case report and review of literature

**DOI:** 10.1016/j.ijscr.2019.03.049

**Published:** 2019-04-05

**Authors:** Hthayyim Khalid Ahmed, Rawa Bapir, Goran Fryad Abdula, Karzan Mohammed Salih Hassan, Rawa Muhsin Ali, Mahabad Abdalaziz Salih

**Affiliations:** a Sulaymaniyah Surgical Teaching Hospital, Urology Department, Sulaymaniyah, Iraq; b Shaheed Shawkat Haji Musheer Hospital, Urology Department, Said Sadiq/Sulaymaniyah, Iraq; c Shar Teaching Hospital, Urology Department, Iraq; d Sulaymaniyah Surgical Teaching Hospital, Department of Surgery, Iraq; e Shorsh General Teaching Hospital, Pathology Department, Iraq; f University of Sulaymaniyah, Faculty of Medical Sciences, School of Medicine, Department of Radiology, Iraq

**Keywords:** Adrenal gland, Adrenalectomy, Adrenal metastasis, Case report, Renal cell carcinoma

## Abstract

**Introduction:**

Renal cell carcinoma (RCC) is known to metastasize to almost every organ. A well-recognized phenomenon in RCC is late metastatic recurrence after nephrectomy which is arbitrarily defined as more than 10 years. The incidence of solitary ipsilateral and contralateral adrenal metastasis is 3% and 0.7% respectively in patients who have underwent radical nephrectomy.

**Case report:**

A57-year-old female presented with an incidental ultrasonic finding of a right adrenal mass 15 years after radical nephrectomy for left side renal cell carcinoma. Further evaluation with abdominal contrast enhanced CT scan revealed a right adrenal mass suggestive of metastatic lesion. The results of laboratory examinations showed the mass to be nonfunctional. Open right adrenalectomy was performed. She was discharged home on 4^th^ postoperative day. Pathological examination revealed morphological and immunohistochemical findings in line with metastatic renal cell carcinoma of the left kidney. During the last 2 years she has being on regular follow up, neither local recurrence nor distant metastasis anywhere have been detected by whole body Positron Emission Tomography(PET) scan.

**Discussion:**

Late metastatic contralateral adrenal recurrence from RCC is very rare and to the best of our knowledge, 5 similar cases have been reported 10 years after radical nephrectomy. Early diagnosis of adrenal metastasis is challenging because they are usually silent both anatomically and functionally.

**Conclusion:**

Solitary contralateral adrenal metastatic recurrence of RCC is extremely rare event. Surgical removal is a wise option in these cases that may improve survival.

## Highlights

• A well-recognized phenomenon in RCC is late metastatic recurrence after nephrectomy.
• Solitary contralateral adrenal metastatic recurrence of RCC is extremely rare.
• Early diagnosis of adrenal metastasis is challenging because they are usually silent.
• This paper is the delayed solitary metastatic recurrence of the renal cell carcinoma to the contralateral adrenal gland.

## 1 Introduction

Renal cell carcinoma (RCC) is known to metastasize to almost every organ. A well-recognized phenomenon in RCC is late metastatic recurrence after nephrectomy which is arbitrarily defined as more than 10 years. This could happen even in early stages of RCC when it has been completely removed. Lungs, abdomen, bones, and brain are among the most common metastatic sites [1,2]. However, isolated adrenal metastasis from RCC is uncommon. The incidence of solitary ipsilateral and contralateral adrenal metastasis is 3% and 0.7% respectively in patients who have underwent radical nephrectomy. Good prognostic signs include an early stage and low grade tumor with a long interval from the diagnosis to the development of metastasis [3]. In line with the SCARE criteria [4]. We here present a case of metastatic recurrence of RCC to the contralateral adrenal gland 15 years after radical nephrectomy.

## 2 Case report

**Fig. 1:**
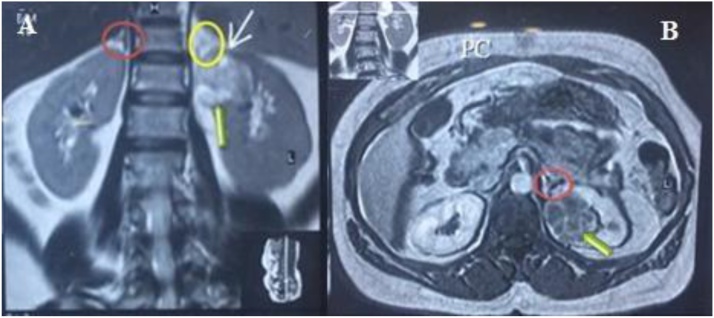
Magnetic resonance imaging, (A) coronal T2W image showing normal both adrenal glands (red circle :Rt adrenal, yellow circle :Lt adrenal), (B) Gadolinium enhanced T1W axial image showing contrast enhanced upper pole renal mass with normal looking Lt adrenal gland.

**Fig. 2:**
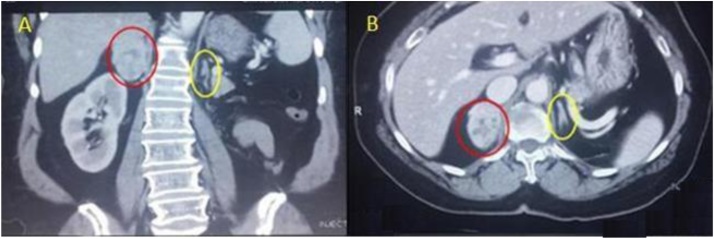
contrast enhanced CT scan of the abdomen. Coronal (A) and axial (B) sections showing an enhanced right adrenal mass (red circle) and normal looking left adrenal gland (yellow circle).

**Fig. 3:**
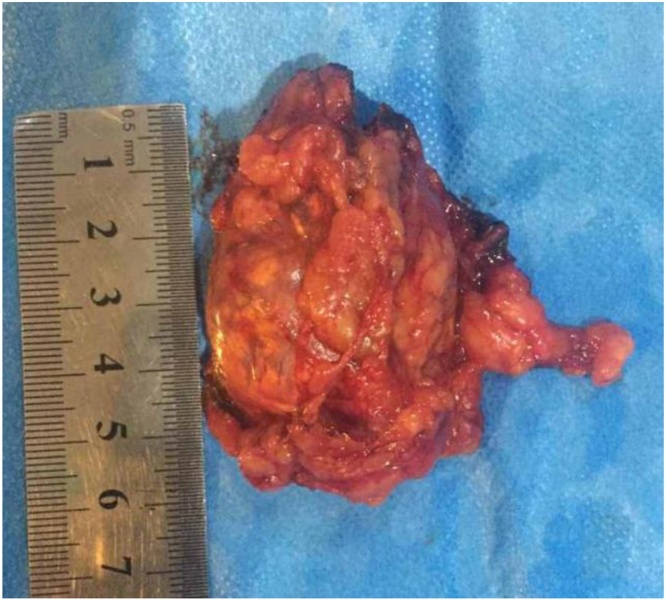
Completely excised right adrenal gland with the mass.

**Fig. 4:**
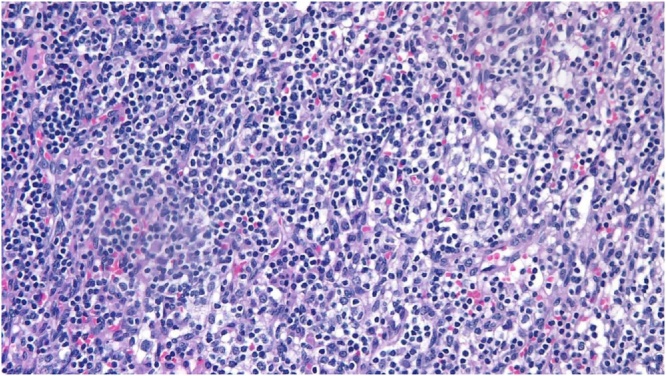
Histologic appearance of clear cell renal cell carcinoma on hematoxylin and eosin stain showing polygonal cells with marked clear cytoplasm and medium-sized nuclei with prominent nucleoli arranged in sheets and tiny clusters.

**Fig. 5:**
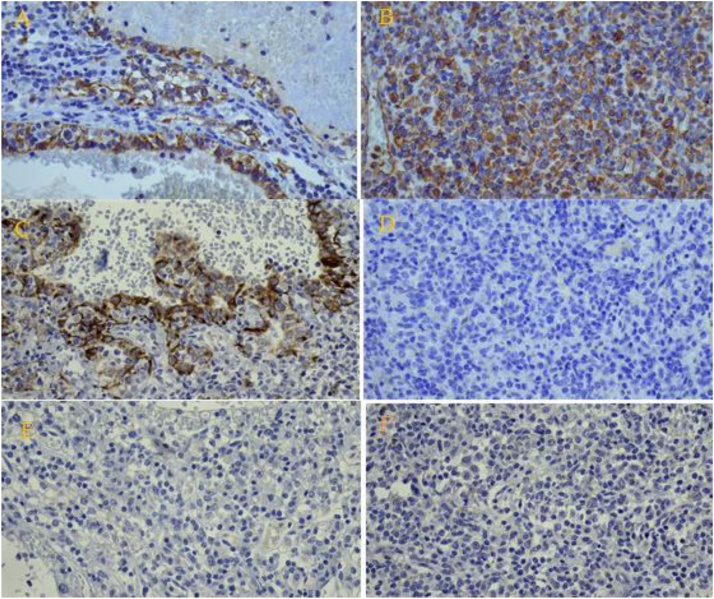
Immunohistochemical examination showing: Positive results for (A) CK7, (B) Vimentin, and (C) AE1/AE3. Negative results for (D) CDX-2, (E) Inhibin, and (F) Synaptophysin.

A 57 -year-old female presented with incidental ultrasonic evidence of left upper pole renal mass in Nov. 2001. Further evaluation with abdominal Magnetic Resonance Imaging revealed a mass in the upper pole of the left kidney with radiologic characteristics of renal cell carcinoma (Fig. 1). Left radical nephrectomy was performed sparing the left adrenal gland. The pathology specimen analysis showed a cystic mass 3 × 3 × 5 cm with yellowish friable tissue. Sections showed malignant epithelial cells, arranged in sheets. The picture was consistent with renal cell carcinoma, Grade II Fuhrman nuclear characteristics, confined to the capsule, neither pelvicalyceal nor vascular invasion was found (pT1bN0M0). Postoperatively she did not receive immunotherapy or chemotherapy. Apart from her hypertension which was well controlled with amlodipine and valsartan, subsequent clinical and radiological follow up showed no local or metastatic recurrence till 5 years after the operation then she stopped her visits. On October 2016 an incidental mass was found in the right adrenal gland during a checkup visit for the status of her right solitary kidney. Abdominal and pelvic computed tomography scan was done, revealing a well-defined mass with a smooth outline in the right adrenal gland measuring 54 × 48 × 39 mm with a central necrosis. The density of the solid component was 38 HU. In dynamic study the solid component showed significant enhancement after intravenous contrast administration (Fig. 2). Thorough hematological, biochemical and hormonal investigations were performed; all were within normal range. The results of laboratory examination showed the adrenal mass to be nonfunctional. The condition was well clarified for the patient and consent was taken to do right adrenalectomy. Under general anesthesia, in left lateral position through right transcostal incision, right adrenalectomy was done (Fig. 3). No any perioperative complications were recorded and she was discharged home on 4^th^ post-operative day. Pathological examination revealed morphological and immunohistochemical findings in line with metastatic renal cell carcinoma, including positive staining for AE1/AE3, cytokeratin 7, vimentin, and CD10, and negative staining for CDX-2, inhibin, and synaptophysin (Fig. 4, Fig. 5). During the last 2 years she has being on regular follow up. Whole body Positron Emission Tomography-Computed Tomography with fluorodeoxyglucose was performed, neither local nor metastatic recurrence was observed in any system.

## 3 Discussion

RCC has the characteristic features of late local and metastatic recurrence. In a large published series of patients with RCC treated with radical nephrectomy, those who survived more than 10 years had 11% chance of late recurrence. Therefore 10 year follow up by imaging is suggested, yet chance of recurrence exists even beyond this timeline, so special care should be maintained in patients with previous history of RCC [2]. Late metastatic contralateral adrenal recurrence from RCC is very rare and to the best of our knowledge, the literature contains only 5 cases similar to our case where metastatic recurrence happened after 10 years of radical nephrectomy (Table 1). This delayed metastatic recurrence can be explained by 2 reasons; First, slow growth of tumors, specifically in case of low grade tumors. Second, in some cases the detection of metastasis will be delayed due to failure of regular imaging follow up. Both of these points were present in our case [5]. Early diagnosis of adrenal metastasis is challenging because they are usually asymptomatic both anatomically and functionally, although on rare occasion the patient may present with symptoms and signs of adrenal insufficiency. So the main means to diagnose these lesions are regular imaging during the follow up period [1]. However it is difficult to determine whether the mass is a primary adrenocortical carcinoma, benign adrenal adenoma or metastasis through imaging studies. The presence of a well vascularized solitary adrenal mass with normal hormonal studies is more suggestive of metastatic lesion rather than primary adrenal mass. In 82% of the reported CAM cases diagnosis was made through CT scan, thus it is the imaging of choice [3]. Similarly in our patient the findings in CT scan were consistent with adrenal metastasis. The only known effective treatment in patients with limited metastasis is surgical removal that improves 5 year survival rate by 29 to 35%. This approach is also amenable to solitary adrenal metastasis [6]. Although laparoscopic technique is regarded as the standard of care in cases of small adrenal mass, however we did open adrenalectomy due to limited experience of laparoscopy at our center.

**Table 1:** Literature summary of contralateral adrenal metastasis from renal cell carcinoma more than 10 years after radical nephrectomy.

reference	Age/sex year	Primary surgery to Metastasis/years	Diagnostic procedures	treatment	Follow up year
Piotrowicz et al. [1]	64/F	17	CT	• Rt adrenalectomy with adrenal vein thrombectomy • Referral to thoracic surgeon for management of single pulmonary metastsis	NS
Kessler et al. [6]	72/M	17.8	CT	adrenalectomy	3.5/alive
Lemmers et al. [7]	68/M	15	CT	Lt adrenalectomy	1/alive
Mesurolle et al. [8]	66/m	23	CT/percutaneous biopsy	Lt adrenalectomy	1/alive
Sagalowsky and Kyle Molberg [9]	63/M	22	CT	Lt adrenalectomy	5.6/alive
The present report	57/F	15	CT	Rt adrenalectomy	2/alive

## 4 Conclusion

Solitary contralateral adrenal metastatic recurrence of RCC is extremely rare event. It can occur late after radical nephrectomy. CT scan with contrast is the imaging of choice that helps in diagnosis of adrenal metastasis. Surgical removal of CAM is a wise option in these cases that may improve survival.

## Conflicts of interest

There is no conflict of interest.

## Funding

None to be stated.

## Ethical approval

Approval has been given by Ethical committee of University of Sulaymanyiah.

## Consent

Written informed consent was obtained from the patient for publication of this case report and accompanying images. A copy of the written consent is available for review by the Editor-in-Chief of this journal on request.

## Author contribution

**Design and idea**: Hthayyim Khalid Ahmed, Rawa Hama Ghareeb Ali, Goran Fryad, Karzan Mohammed Salih Hassan and Mahabad Abdalaziz Salih.

**Drafting:** Rawa Hama Ghareeb Ali, Rawa Muhsin Ali and Goran Fryad.

**Data collection**: Rawa Hama Ghareeb Ali, Rawa Muhsin Ali.

**Final revision**: Hthayyim Khalid Ahmed, Rawa Hama Ghareeb Ali, Goran Fryad, Mahabad Abdalaziz Salih, and Karzan Mohammed Salih.

## Registration of research studies

Not applicable.

## Guarantor

Rawa Hama Ghareeb Ali.

## Provenance and peer review

Not commissioned, externally peer-reviewed.
